# Exploring the Frontiers of Ovarian Tissue Cryopreservation: A Review

**DOI:** 10.3390/jcm13154513

**Published:** 2024-08-02

**Authors:** Tia Ramirez, MaryEllen Pavone

**Affiliations:** 1 McGaw Medical Center, Northwestern University, Chicago, IL 60611, USA; 2 Department of Obstetrics and Gynecology, Division of Reproductive Endocrinology and Infertility, Northwestern University, Chicago, IL 60611, USA

**Keywords:** ovarian tissue cryopreservation, in vitro maturation, in vitro follicle maturation, live birth outcomes

## Abstract

**Objective**: This paper serves as an up-to-date narrative review of the most effective methods and outcomes of ovarian tissue cryopreservation (OTC) with new data comparing this method to oocyte and embryo cryopreservation as well as its utility in restoration of endocrine function. **Background**: Data on OTC are becoming more available as more patients are achieving cancer remission and choosing to use their cryopreserved tissue to conceive or restore endocrine function. With OTC only recently becoming a non-experimental method of fertility preservation, it is important to evaluate, compare, and optimize current practices to improve live birth outcomes. **Methods**: A literature search of meta-analyses, systematic reviews, case series, retrospective studies, and randomized control trials was performed using the PubMed database with multiple search terms. **Discussion**: Current practices and outcomes of OTC remain heterogeneous, though they are becoming more streamlined with the emerging data on successful live births. Multiple aspects of OTC have been studied to optimize protocols, particularly methods of cryopreserving, in vitro maturation, and transplantation. In vitro follicle maturation is a novel application with emerging data on methods and outcomes. OTC is a versatile method not only for fertility preservation but also for hormone restoration as well. With wider usage of OTC, ethical dilemmas will need to be addressed. **Conclusions**: OTC can be used as fertility preservation for a variety of patients. Recent studies suggest it may be comparable to embryo cryopreservation, but with growing data on live births, comparative studies should continue to be performed. In vitro follicle maturation (IVFM) is a promising application of ovarian tissue harvesting. Data are lacking on cost-effectiveness, patient satisfaction, and morbidity associated with OTC.

## 1. Introduction

Ovarian tissue cryopreservation (OTC) has been offered to patients for over 20 years and is increasingly leading to live births [1,2]. OTC does not require extensive medication regimens or preparations, which can be appealing to patients that do not want to, or cannot, delay chemotherapy. Additionally, because there is no need to undergo ovarian stimulation, OTC can be performed in patients with hormone sensitive cancers or prepubertal girls [3]. For prepubertal girls, OTC is currently the only available fertility preservation method [4]. Although OTC is an option for oncology patients, there are concerns with offering it to patients with breast or ovarian cancers and lymphomas or leukemias, given the potential risk of re-introducing malignant cells with the re-implantation of the cryopreserved tissue [5].

Although OTC has largely been studied in patients with malignancies, as more data become available, OTC may be considered for other indications [6]. Although data are still sparse relative to other methods of fertility preservation, OTC is a desirable and effective method and should be offered as an established option for fertility preservation [2,4,7]. Historically, OTC was considered investigative; however, the American Society for Reproductive Medicine recently issued a committee opinion stating OTC is no longer experimental and can be used as a reliable method to achieve pregnancy [8]. Furthermore, the European Society of Human Reproduction and Embryology (ESHRE) recommends offering OTC in patients undergoing moderate to high risk gonadotoxic treatment where oocyte stimulation and harvesting is not feasible, or per patient preference [6]. The release of these statements increases the likelihood that more providers will begin offering this option to patients.

OTC is a growing field with multiple applications. Currently, research focuses on improving live birth outcomes by optimizing maturation and growth of both follicles and oocytes. With OTC becoming more popular, it is important to optimize these protocols to continue to increase the likelihood of live births. Additionally, other applications of cryopreserved ovarian tissue are being studied such as its use in restoration of endocrine function and long-term health outcomes in these patients. This paper highlights the more recent developments and insights regarding methods, applications, and outcomes of OTC with newer data comparing outcomes to oocyte and embryo cryopreservation.

## 2. Methods

**Figure 1:**
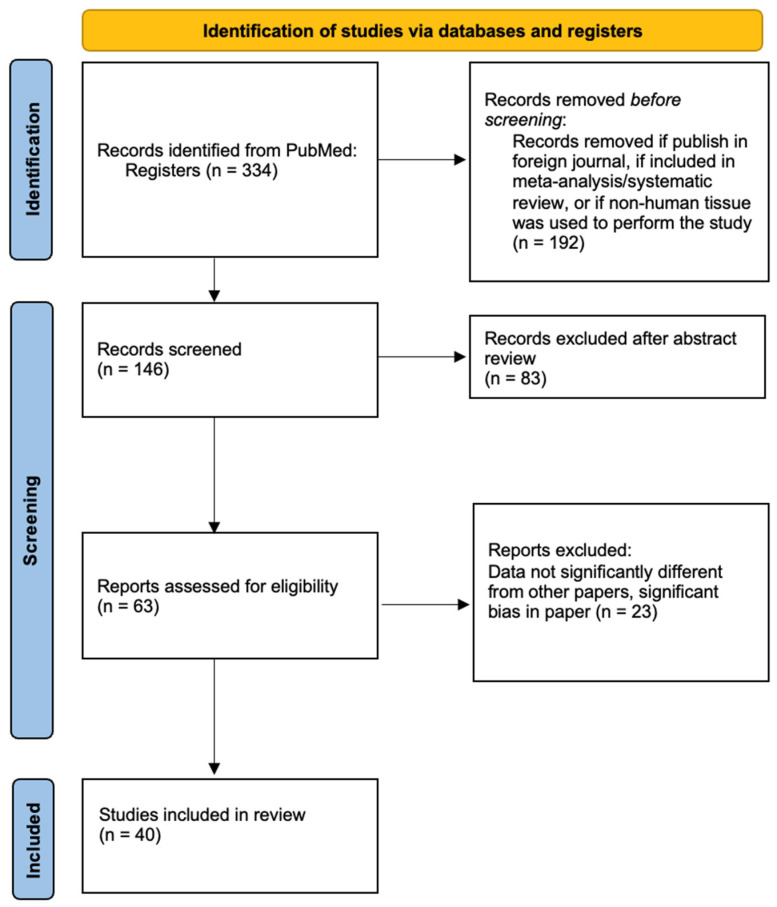
Flow-diagram of source selection.

A literature search of meta-analyses, systematic reviews, retrospective studies, case series, and randomized control trials on PubMed was performed for this narrative review. Search terms used included: “Ovarian tissue cryopreservation”, “ovarian tissue transplantation”, “in-vitro follicle maturation of ovarian tissue”, “endocrine restoration”, and “patient perspectives”. Inclusion criteria were articles published from January 2010 to March 2024, English language, and use of human oocytes. Articles were excluded if the primary focus was oocyte or embryo freezing, if the study included patients with only one type of cancer, if the study focused on a single aspect of ovarian tissue cryopreservation (i.e., tissue size, delivery time of tissue samples), or if the article was included in one of the meta-analyses included in this review (Figure 1).

## 3. Cryopreservation

Much like oocytes and embryos, ovarian tissue can be cryopreserved and stored or transferred fresh. As most patients undergoing OTC plan to use their autologous tissue and will need time to undergo chemotherapy, it is most common to cryopreserve tissue after it is harvested in patients diagnosed with cancer. Given OTC’s relative novelty, there is no consensus on the most effective methods of harvesting or cryopreservation, although there are data available that begin to compare the different methods currently in place. Specifically, the optimal amount of tissue and surgical technique remain heterogeneous. A systematic review by Diaz et al. found that harvested tissue size may be correlated with pregnancy and live birth outcomes; however, no standard protocols are in place [9]. Additionally, preliminary animal studies of whole ovary harvesting are being done with promising findings, but this has not yet been performed in humans [10]. Regardless, nearly all fertility centers remove the medulla from the ovarian cortex and cut the tissue into small strips prior to exposure to the freezing medium as majority of the immature oocytes and primordial follicles reside in the cortex. Additionally, this maintains the most homeostatic paracrine environment when thawed [11].

Slow freezing is the traditional method used to cryopreserve most tissues. With this method, the tissue is placed in a low concentrate cryoprecipitate followed by a programmable freezing device to slowly reach a freezing point. Vitrification is the process of rapid freezing in a high concentrate cryoprecipitate and has been most effective at preventing creation of ice crystals inside cells. Vitrification allows for less time preparing the tissue, is lower cost, and in some studies has been shown to lead to improved tissue function; however, both freezing methods remain in use [12,13]. A meta-analysis of 15 studies performed by Behl et al. compared the proportion of intact primordial follicles after slow freezing and vitrification as a measure tissue integrity and found no significant difference between vitrification and slow freezing (RR = 0.89; 95% CI, 0.74, 1.09) [14]. A few studies, including a large meta-analysis, have performed sub-analyses on vitrification compared to slow freezing and have shown that both methods may lead to comparable clinical pregnancy and live birth outcomes [13,15]. A limitation to both slow freezing and vitrification is the potential for oxidative damage to the cells. This damage can be partially mitigated by the addition of antioxidants to prevent creation of reactive oxygen species [16]. Larger studies comparing the two methods are needed to determine which method is the most effective.

Multiple methods to harvest ovarian tissue are in practice and involve creating small pieces of the ovarian cortex prior to freezing. There are no data on optimal tissue sample size. Additionally, both slow freezing and vitrification remain viable methods for OTC, and neither has been shown to lead to significantly improved outcomes regarding live births.

## 4. Transplantation

Whether fresh or thawed, ovarian tissue is transplanted to the patient either orthotopically or heterotopically. In women with remaining ovarian tissue, the preserved ovarian tissue can be re-implanted near the site of the remaining tissue. Other sites considered to be orthotopic include the abdomen near the anatomic ovarian site such as the abdominal rectus muscle, peritoneum, abdominal wall, the ovarian ligaments, ovarian cavity, and fallopian tube [16]. Heterotopic transplantation is typically in the arm. There is potential for live births despite the implanted tissue’s heterotopic location as it is thought that the transplanted tissue may provide sufficient endocrine function to restore native ovarian function, so long as the patient has not experienced complete ovarian insufficiency from their treatment [4]. However, heterotopic transplantation has not been shown to be as effective as orthotopic transplant regarding pregnancy outcomes (23% vs. 3%), though studies are limited by a small number of heterotopic transplants [17].

Another important aspect to consider when transplanting thawed ovarian tissue is that there is a high risk of follicle loss in the postoperative phase. One study showed that only a small percentage of follicles (7%) were lost during freezing and thawing, while a much larger proportion (68%) were lost during the ischemic phase of tissue re-vascularization after transplantation [18]. Many fertility specialists have been studying ways to mitigate this issue, including the best operative technique and agents that could be used to maintain the integrity of the tissue. Regarding technique, Silber has the most well described method to perform a successful tissue transplant regardless of using fresh or thawed tissue [19]. Ensuring vascular integrity is the focus of each of the steps. The cortex of the harvested ovarian tissue is first trimmed down to 0.75–1.0 mm thickness to promote rapid re-vascularization. Next, hemostasis of the graft bed is assured using bipolar energy and then reinforced with nylon interrupted sutures to prevent hematoma formation under the graft. The grafts are then implanted to the highly vascular ovarian medulla and continual pulsatile irrigation with heparinized saline is applied to the graft surface to prevent adhesion formation [19]. Other methods to mitigate follicular loss is the use of a pro-vascularizing agent such as Sphingosine-1-phosphate (S1P) [1]. Large studies have not yet been conducted, but S1P shows promise in that oocyte stimulation in patients who have been transplanted with S1P have produced more mature oocytes than those without [1].

It is important to note that multiple cohort studies have reported spontaneous conception after ovarian tissue re-implantation. Additionally, oocyte stimulation with subsequent ICSI or IVF were successfully performed with the re-implanted tissue. Dolmans et al. performed a review of 285 patients out of five European centers [20]. A total of 167 of these women chose to attempt spontaneous conception. Of those women, 40% became pregnant (n = 67), and 30% (n = 52) had live born infants. In addition, 109 of the women attempted IVF with their transplanted tissue, and 36% (n = 39) conceived with 21% (n = 23) having live born infants [20]. This review demonstrates the great potential OTC has for restoring fertility for women with primary ovarian insufficiency and further supports the decision to declare OTC as no longer experimental.

Similar to ovarian tissue harvesting, there is no gold standard protocol for re-implantation. Silber has the most well-defined protocol, though not all fertility centers use this method. The site of re-implantation is important for potential spontaneous and assisted-reproductive live birth outcomes. It is important to consider loss of follicles when transplanting tissue and further studies will need to be done to assess the use of additives or supplements to mitigate these loses. Despite the length of cryopreservation and age of patient at the time of tissue harvesting, spontaneous conception is possible.

## 5. In Vitro Follicle Maturation (ivFM)

Girls and women with blood-born leukemias, ovarian cancers, or other cancer with a high risk of ovarian metastasis may not be suitable for re-transplantation of ovarian tissue because it carries risks of the cancer being reintroduced [21]. Additionally, many patients undergoing OTC are pre-pubertal, thus their tissue is thought to contain only primordial follicles. It is possible to achieve spontaneous conception with re-implantation, but there is also potential to stimulate growth of follicles in vitro [22]. In cases where whole ovarian tissue should not be transplanted, multiple methods are being developed to try and mitigate the potential harm of re-introducing cancer cells such as ivFM using scaffolds to mature pre-antral follicles, eradication of malignant cells, stem cell oogenesis, and maturation of oocytes [23]. ivFM is being studied [21] as a process that helps with the maturation of the primordial follicle into the cumulus-oocyte complex. Subsequent in vitro maturation is then performed on the immature oocyte followed by intra-cytoplasmic sperm injection (ICSI) to create embryos, thus eliminating the need to re-implant ovarian cortical tissue.

This ivFM process is typically initiated with the ovarian cortex still intact to promote the optimal growth environment as both stimulating and inhibitory factors present in the tissue are difficult to replicate using culture media [21,23,24,25]. For initial follicle growth, the optimal media is still being developed; however, specific additives (glucose, insulin, FSH, antibiotics, antioxidants, AMH, etc.) are required for maturation [24]. The inter-place between oocytes and granulosa cells seems to be a key factor in successful growth and maturation, specifically the inactivation of the Hippo and PI3K/AKT pathways, which are still not fully understood [24]. A newer method of follicle maturation has been shown to be effective and is accomplished by separating the follicle from the ovarian cortex followed by placement in a follicle culture system. The culture medium that has been shown to produce the most meiotically competent oocytes consists of a two-step culture method using a 3D matrix: 0.5% alginate hydrogels followed by low-attachment plates [24,26]. In one study, 20% of the dual cultured follicles produced meiotically competent cells (relative to <10% of the single media follicles). What is most interesting is that this was achieved despite the antral follicle never reaching an appropriate pre-ovulatory size of 15–20 mm [26]. Maturation rates of oocytes harvested from matured follicles are much less than maturation rates achieved with IVF; however, live births have been reported [21,27].

Multiple methods are being investigated to try and mitigate the potential harm of re-introducing cancer cells with OTT such as ivFM. Aspects of folliculogenesis such as maintaining follicles within the ovarian cortex have been proven crucial to the initial growth of follicles, but much remains unknown. Few multi-step culture systems have been promising and continue to be studied. Thus far, ivFM has demonstrated no increased risk of genetic abnormalities or cancer in offspring, though the available studies involve less than 50 cases [22].

## 6. In Vitro Maturation (IVM)

In vitro maturation (IVM) is the method of maturing oocytes to the MII stage either with oocytes isolated from the ovarian medulla or following isolation of oocytes from mature follicles achieved with ivFM [28]. Two protocols have been studied on oocytes from harvested ovarian tissue: monophasic and biphasic. Monophasic IVM is carried out in IVM medium supplemented with highly purified human menopausal gonadotropin, human chorionic gonadotropin, and human serum albumin whereas biphasic IVM is supplemented with recombinant follicle stimulating hormone (rFSH), insulin, estradiol, human serum albumin, and c-type natriuretic peptides. Additionally, the biphasic method includes a second IVM medium with rFSH, insulin, estradiol, and human recombinant amphiregulin [11].

A single study comparing these two methods in a small cohort of patients was published by De Roo et al. in 2021 [11]. Oocytes were harvested from medullary tissue and were matured either monophasically or biphasically as described above. Oocyte maturation rates for monophasic vs. biphasic media were 35% and 56%, respectively. Furthermore, fertilization rates are 68.4% vs. 80% [11]. Matured oocytes can additionally be cryopreserved for use with future intracytoplasmic sperm injection (ICSI). When ICSI is performed, 24% of monophasic IVM oocytes vs. 38% of biphasic IVM oocytes achieve good quality day 3 embryos. Both methods showed increased live birth rates (from 20–35%) when compared to oocytes that have not undergone IVM [11]. There is potential for oocytes derived from ivFM to undergo IVM with ICSI thereafter; however, minimal studies have been reported and from the limited data available, it appears oocytes derived from IVFM respond differently than those obtained from the medulla [25].

IVM is a more established method used on ovarian tissue. Multiple studies have shown positive live birth outcomes and methods are much more streamlined than those for ivFM. Much like ivFM, the IVM process has proven to be difficult as well as technically demanding [6]. More studies must be done to compare live birth outcomes of ivFM matured oocytes versus medullary oocytes as well as cost-analyses of these methods.

## 7. Endocrine Restoration

Other more experimental applications of OTC have been used in the peri- and post-menopausal period as anti-aging and hormonal treatment since transplanted ovarian tissue has been found to have intact endocrine function [29]. Most studies have shown that OTC preserves the tissues’ ability to stimulate and respond to the hypothalamic-pituitary-ovarian axis to produce menstrual cycles. Knowing this, transplantation of OTC could theoretically treat the hypo-estrogenic effects of menopause, potentially improving bone, cardiovascular, and mental health as well as hot flashes [30]. This would be a separate utility from fertility preservation and may appeal to a broader demographic than the current patients that pursue OTC.

The optimal age to harvest ovarian tissue is by the age of 25 to have enough reserve to provide endocrine function [31]. Studies have shown endocrine function of transplanted tissue up to seven years, and it is postulated that transplantation may be able to be repeated once endocrine function dwindles [31]. A major supporting argument for ovarian tissue transplantation (OTT) for endocrine function is that this method may lead to estrogen secretion at a physiological, lower, and safer concentration than conventional hormone replacement therapy (HRT). It is well known that naturally produced hormones generally have lower risk profiles (i.e., less risk of deep vein thrombosis) adding further benefit to this application.

The largest meta-analysis performed on endocrine outcomes in patients with transplanted ovarian tissue was performed by Khattak et al. Return of endocrine function was measured by serum levels of estrogen and FSH. Pooled means for pre-transplant estrogen was 101.6 pmol/L, which increased post-transplant to 522.4 pmol/L. The pooled mean of pre-transplant FSH was 66.4 IU/L, which decreased post-transplant to 14.1 IU/L. The median time to return of FSH to a value <25 IU/L was 19 weeks. In a subset of the population studied, 72% of patients had return of menses after transplantation with average onset of menses 18 weeks post-transplant. The median duration of graft function was 2.5 years (range: 0.7–5 years) [13].

An additional cohort study out of Bologna on tissue obtained from 1026 patients was performed. Only a small sample (2.3%) elected ovarian tissue transplantation; however, return of menstruation occurred in 88% of those who had a pre-transplant clinical diagnosis of primary ovarian insufficiency. What else was fascinating was that in patients who had undergone at least one cycle of chemotherapy prior to ovarian tissue harvesting, they had maintained endocrine function of their ovarian tissue when it was re-implanted years later. This study also found that the integrity of endocrine function was maintained in tissue that had been cryopreserved for up to 15 years [32].

OTT for endocrine function is an emerging concept. The negative health effects hypo-estrogenism has on the female body are well known, and treatments continue to be developed. Regarding OTT, data on menopausal symptoms and bone health post-transplant are scarce; however, with estrogen levels that achieve return of menses, it can be assumed that there is likely to be benefit to both. Given the fewer side effects of OTT than traditional HRT, there is potential in this application, though costs remain much higher than HRT [31].

## 8. Patient Outcomes and Case Studies (Appendix A)

Data on pregnancy and endocrine function in patients who have undergone ovarian tissue preservation and transplantation are becoming more available. A retrospective analysis out of Europe studying 74 patients demonstrated 21 pregnancies and 17 live births within 1 year of orthotopic tissue transplantation [33]. The specifications on slow freeze versus vitrification were not delineated. None of the harvested follicles underwent ivFM, but medullary oocytes underwent IVM of some sort. Culture media were not specified nor were they unified.

A retrospective cohort study of 77 patients out of Belgium was performed to evaluate live birth rates after IVM of 1220 medullary oocytes. Oocyte maturation rates were 39%. Of these mature oocytes, a subset of approximately 82 oocytes (13 patients) underwent ICSI for embryo freezing. Approximately 25% of the oocytes were successfully fertilized and vitrified. Only 7 of the 13 patients with embryos, and 5 of the 64 patients with cryopreserved IVM oocytes returned for transfer. For one of the five patients with cryopreserved oocytes, the tissue was thawed and then underwent ICSI. The embryo was transferred fresh and resulted in an uncomplicated live birth. For the patients that froze embryos, two had uncomplicated live births resulting from thawed embryos [34].

Another study of one hundred and nine females was conducted in the United States and demonstrated promising outcomes. Women between ages 6 and 35 were referred for possible OTC over a 20-year period, with either slow freeze or vitrification. Thus far 13 patients have returned up to 18 years later to have their tissue transplanted back. All 13 patients had return of ovarian function within 5 months post-transplant with regular menstrual cycling and remained functional for up to 5 years or longer. In addition, 10 of the 13 (77%) became spontaneously pregnant at least once, resulting in 13 healthy babies [15].

In a meta-analysis of 19 studies comparing live birth outcomes of 181 women who received ovarian transplants, including frozen–thawed transplants or fresh donor grafts, the live birth rate for frozen transplants was 28% (95% CI: 24–34%) [13]. Regarding outcomes for fresh transplants (n = 45), nearly all of the fresh allograft donors and recipients were twins with one twin having either idiopathic or iatrogenic premature ovarian insufficiency. The live birth rate for fresh transplants was noted to be 45% (95% CI: 23–86%) [13,19]. The discrepancy between fresh and frozen transplant outcome data is not only because of the small data set of fresh transfers but likely also because viable tissue may be lost during both the cryopreservation and thawing processes. This tissue loss can only be partially mitigated by cryoprotectants and antioxidants, whereas fresh tissue transfers would not undergo temperature changes [18]. This meta-analysis also demonstrated the median duration of graft function to be 2.5 years [13]. Newer studies demonstrate potential function up to 7 or 8 years in some cases [19].

OTC has been expanded to broader patient populations including patients with Turner syndrome (TS). Spontaneous pregnancy rates in women with TS is around 5.6% with most pregnancies occurring in women with mosaic TS and only 0.4% of spontaneous pregnancies in women with a non-mosaic karyotype. This makes TS patients great candidates for ovarian tissue harvesting followed by ivFM or IVM or donor ovarian tissue transplantation. Patients with TS would need to undergo OTC early as their follicle counts decrease much earlier than in patients without TS [35].

A new meta-analysis was published comparing live birth rates between ovarian tissue, oocyte, and embryo cryopreservation. The sample size of 3271 patients was heterogeneous; however, all patients underwent chemo-radiation. Results showed live birth rates were 27%, 8.76%, and 6.74% for oocyte, ovarian tissue, and embryo cryopreservation, respectively. These rates are raw percentages based on number of live births divided by number of patients who used each method of cryopreservation. Studies show very few people return to use their cryopreserved tissue, particularly ovarian tissue, which profoundly confounds this data. Differences are also believed to be heavily skewed by the fact that most patients that undergo OTC are pre-pubertal, requiring ivFM, IVM, or significant time for their re-implanted tissue to mature [36]. This study was interesting and demonstrates that live births are possible with each method; however, there are significant limitations when interpreting this data given how few patients have returned to use their cryopreserved tissue. Additionally, methods of cryopreservation and use of IVM were not reported. An additional meta-analysis by Fraison compared live birth per pregnancy rates and showed LBR after frozen-thawed embryo transfer is 41%, IVF of thawed oocytes 32%, IVF after OTT 19% vs. spontaneous pregnancy after OTT 33%. Nearly 100% restoration of endocrine function was also noted after OTT [37].

## 9. Discussion: Limitations and Future Directions

OTC is becoming a reliable option for all patients undergoing fertility preservation. Again, OTC has historically been an emergent method of fertility preservation as it originally did not have well known pregnancy and live birth outcomes. Now that data are becoming available, and methods to harvest, cryopreserve, and re-implant tissue are leading to increasingly more positive fertility outcomes, OTC has the potential for becoming a more desirable method to preserve fertility in the general population. Certain aspects of OTC lead to more favorable outcomes, such as ovarian tissue removal prior to chemotherapy exposure, slow freezing, oocyte maturation, and orthotopic transplantation; however, what is most notable about advances regarding OTC is the potential that lies in in vitro follicle maturation and subsequent oocyte maturation. This technology holds the possibility of becoming as effective as traditional IVF as well as diminishes the possibility of re-implanting malignant cells in onco-fertility patients [27].

Two meta-analyses comparing multiple fertility preservation options show significantly lower live birth outcomes for OTC compared with IVF with oocyte or embryo cryopreservation but are limited by many patient factors and data gaps. Epidemiological studies showed that women who choose elective egg or embryo freezing are commonly Caucasian, between 36 and 40 years of age, with higher education, and professional employment, which is a much different demographic than those currently undergoing OTC [38]. Appropriate FP counseling based on the literature on live birth outcomes should be given to women at the time of diagnosis to assist with their decision.

Great vulnerability due to the anxiety of a cancer diagnosis and limited time for discussion due to the start of treatment affects patients’ decision making; however, providers must always provide full informed consent and consider patient autonomy. A large barrier is that fertility preservation is simply not discussed for nearly 50% of patients at initial diagnosis [22]. Health professionals are often reluctant to initiate fertility preservation discussions or simply do not have the proper tools to initiate that conversation. This is due to multiple factors but leans on the importance of having a multi-disciplinary team including patient navigators. Assisting patients with this decision, regardless of the reason for cryopreservation, likely impacts their satisfaction and improves their quality of life long-term [8].

With the new data and advances in the field of ovarian tissue cryopreservation, ethical dilemmas will continue to arise, particularly that of management of tissue of deceased patients or unused tissue [39]. Ethical guidelines must be established to ensure proper use of tissue. Additionally, with many patients being pre-pubertal, ethical committees can serve to assist parents with decision making in addition to moderating the reasoning behind these decisions. OTC continues to prove successful at preserving fertility in addition to its potential to restore endocrine function. The methods to do so continue to improve and transform as more patients choose OTC. As OTC becomes applied to a broader population, ethical guidelines should be developed and studies comparing cost, patient satisfaction, morbidity, and live birth outcomes of ivFM/IVM relative to traditional oocyte cryopreservation and in vitro fertilization should be published.

## Appendix A

**Table A1:** A summary of case reports and meta-analyses included in this review.

Authors	Date of Publication	No. of Patients	Outcomes	Methods	Limitations
Khattak et al. [8]	Feb. 2022	547 women	Pooled rates for frozen-thawed ovarian tissue were: 37% (95% CI: 32–43%) for pregnancy, 28% (95% CI: 24–34%) for live birth and 37% (95% CI: 30–46%) for miscarriage. 45 fresh transplants: pregnancy rate 52% (95% CI: 28–96%) live birth rate 45% (95% CI: 23–86%), miscarriage rate 33% (95% CI: 13–89%). Pregnancy rates for slow freezing was 37% as compared to vitrification, which was 44%. 69% pregnancies were conceived naturally, whereas ART was used for 31% of pregnancies.	Meta Analysis of MEDLINE, EMBASE, CINAHL and Cochrane Central Register of Controlled Trials from database inception to October 2020. Studies that reported fertility or endocrine outcomes from either fresh or frozen–thawed ovarian transplants for at least one participant were included. Commentaries, editorials, correspondence and letters were excluded.	Study sizes, very small number of fresh transplants relative to frozen thawed, not all women were tested for menopausal status prior to transplant
Kometas et al. [12]	Dec. 2021	9 biochemical studies	Early clinical outcomes appear favorable for vitrification. However, even if research corroborates conclusions of no clinical or biochemical difference between cryopreservation methods, the decreased costs and increased efficiency associated with vitrification make this method more accessible and cost-effective.	This literature review evaluates clinical and lab-based studies published between January 2012 and June 2020 to determine whether vitrification, the optimal technique for oocyte and embryo cryopreservation, preserves ovarian tissue more effectively than slow freezing	Additional studies and longer term follow-up are needed to establish the efficacy of vitrification
Mohd Faizal et al. [25]	2022	12 studies 590 Females	5724 immature oocytes retrieved via aspiration from visible follicles with needle or using scalpel before ovarian cortex dissection. Following IVM, 1937 oocytes reached MII with an oocyte maturation rate of 33.84%. Afterward, 1169 oocytes were successfully cryopreserved for use in future ICSI/IVF.	Relevant studies within 20 years (2000–2020) were thoroughly searched in EMBASE, PubMed, clinical.gov, and more. IVM as intervention following ovarian tissue harvesting in women with cancer, and oocyte maturation rate as the primary outcome. In one study, OTC was performed after oocyte retrieval following stimulated cycles (ST). Otherwise, all other OTCs were performed in non-stimulated (NS) ovaries. All included studies utilized single-cycle IVM, except for one study that performed two cycles of IVM	The adjunct medium or hormone added to enhance IVM and pregnancy outcomes were not discussed. The participants in this review are mostly post-pubertal; thus, evidence for the pre-pubertal group is limited.
Telfer EE, Andersen CY [21]	May 2021	Review Paper	These studies provided proof of concept that complete oocyte development can be achieved in vitro, and this has driven the development of culture systems that could be applied. Each stage of follicle development, from activation of primordial follicle growth through to maturation, requires changing conditions and so a dynamic culture system is needed. To achieve activation, primordial follicles need to be maintained within small pieces of ovarian cortex containing stromal cells	using culture techniques that support complete in vitro growth and maturation (IVGM) of cryopreserved primordial oocytes into fertile metaphase II (MII) oocytes [11,12] or by harvesting immature oocytes from small antral follicles released during the procurement of the tissue with subsequent in vitro maturation (IVM) and in vitro fertilization (IVF)	Not statistical analyses performed
Silber et al. [15]	Dec 2018	108 women; 13 underwent transplantation; 11 fresh transplants	Ninety-two (85%) of these women underwent unilateral oophorectomy and cryopreservation. Thirteen of the 92 ovary freeze cases (14%) have come back to have their ovary tissue transplanted back. Of those 13, the four most recent cases had been cryopreserved by vitrification, and the other 9 had been frozen by slow freezing. Additionally, there have been 11 fresh transplants either between identical twins or allografts.	Case series	This is a relatively small series compared to the impressive experience of the Belgian, Israeli, Spanish, and Danish centers
Silber et al. [19]	Nov. 2010	9monozygotic twin pairs, 16 cancer patients	All nine returned to regular menses and ovulatory cycles by 60–130 days after surgery. Six of the eight who had normal fallopian tubes delivered eight healthy babies after natural conception, and 4 patients had one early miscarriage each. Fresh 91.9%, Vitrification 89.1%, slow-freeze 41.7%	Case series from a tertiary referral community hospital.	Does not directly compare fresh vs. frozen transplant outcomes. Small sample size. Limited to one center with singular perspective on how to perform harvesting and transplantation
De Roo et al. [11]	Oct 2021	10 women Monophasic OTO-IVM (n = 96 COCs) vs. biphasic OTO-IVM (n = 105 COCs)	Monophasic IVM-oocyte maturation rate 30–40%, fertilization rate by ICSI 35–65%, 5 live births. Biphasic IVM maturation rate: 56%, fertilization rate 80%, 0 live births	A literature search of PubMed with no date restriction. Biphasic IVM was originally studied in high responders (PCOS), only recently being applied to harvested ovarian tissue	Mostly studied in oocytes retrieved in vivo. Only been studied in a very small cohort. No long-term fetal/childhood data.
Vander Ven et al. [28]	Aug 2016	49 women	49 women with a follow-up >1 year after transplantation, the ovaries were active in 67% of cases and the pregnancy and delivery rates were 33 and 25%, respectively. The success rates were age dependent with higher success in women who cryopreserved at a younger age.	Retrospective analysis of transplantation that was performed in 16 centers and data were transferred to the FertiPROTEKT registry.	The cryopreservation and transplantation techniques used have changed during the study period. Post-menopausal status was not confirmed in all patients prior to transplantation
Dolmans et al. [20]	May 2021	285 women	The live birth rate, although not significantly different, was higher in women conceiving naturally (30%) than in those undergoing IVF (21%) Analyzing the number of infants born from each surgical technique, the rates were 30.5% with OTT to the ovary, 34.8% with OTT to the peritoneum, and 34% with the combined technique, indicating that the different sites are equivalent with respect to reproductive efficacy	The current multicenter series includes patients who underwent OTT by one of five teams: Andersen’s team from Denmark (62 patients), Diaz’s team from Spain (53 patients), Donnez and Dolman’s team from Belgium (29 patients), Poirot’s team from France (53 patients), and the FertiPROTEKT network group from Germany, Switzerland, and Austria (88 patients).	These patients presented with failure of IVF treatment, and it was decided to boost their ovarian reserve by OTT. Limited to Europe (different patient demographic, different healthcare system).
Xiao et al. [23]	Nov. 2015	44 patients	Compared to follicles cultured in the alginate hydrogels, follicles cultured using this two-step strategy had significantly greater terminal diameters starting on day 20. After release from alginate encapsulation, follicles had significantly higher hormone levels, indicating that the two-step culture strategy permits an increase in follicle hormone production. Follicles cultured only with alginate encapsulation produced oocytes that either remained in the germinal vesicle stage or degenerated. In contrast, 20% (4 out of 20) of follicles cultured using the two-step strategy produced meiotic competent MII oocytes that extruded the first polar body.	Human follicles were encapsulated and cultured in 0.5% alginate hydrogels. A portion of follicles were then released from the alginate hydrogels at the antral stage, and then continued the cultures in low-attachment plates for up to 40 days.	Study size, technology used is likely expensive and not available in many settings. No data on pregnancy on live birth outcomes.

## References

[1] Marin L., Bedoschi G., Kawahara T., Oktay K.H. (2020). History, Evolution and Current State of Ovarian Tissue Auto-Transplantation with Cryopreserved Tissue: A Successful Translational Research Journey from 1999 to 2020. Reprod. Sci..

[2] Gellert S.E., Pors S.E., Kristensen S.G., Bay-Bjørn A.M., Ernst E., Yding Andersen C. (2018). Transplantation of frozen-thawed ovarian tissue: An update on worldwide activity published in peer-reviewed papers and on the Danish cohort. J. Assist. Reprod. Genet..

[3] Oktay K., Harvey B.E., Partridge A.H., Quinn G.P., Reinecke J., Taylor H.S., Wallace W.H., Wang E.T., Loren A.W. (2018). Fertility Preservation in Patients With Cancer: ASCO Clinical Practice Guideline Update. J. Clin. Oncol..

[4] Pampanini V., Hassan J., Oliver E., Stukenborg J.-B., Damdimopoulou P., Jahnukainen K. (2021). Fertility Preservation for Prepubertal Patients at Risk of Infertility: Present Status and Future Perspectives. Horm. Res. Paediatr..

[5] Khattak H., Woodman H., Afifi Y., Amorim C.A., Fishel S., Gallos I., Coomarasamy A., Topping A. (2022). Experiences of young girls and women undergoing ovarian tissue cryopreservation: A systematic review and thematic synthesis. J. Psychosom. Obstet. Gynaecol..

[6] Anderson R.A., Amant F., Braat D., D’Angelo A., Chuva de Sousa Lopes S.M., Demeestere I., Dwek S., Frith L., Lambertini M., ESHRE Guideline Group on Female Fertility Preservation (2020). ESHRE guideline: Female fertility preservation. Hum. Reprod. Open.

[7] Donnez J., Dolmans M.M., Diaz C., Pel-licer A. (2015). Ovarian cortex transplantation: Time to move on from experimental studies to open clinical application. Fertil. Steril..

[8] Practice Committee of the American Society for Reproductive Medicine (2019). Fertility preservation in patients undergoing gonadotoxic therapy or gonadectomy: A committee opinion. Fertil. Steril..

[9] Diaz A.A., Kubo H., Handa N., Hanna M., Laronda M.M. (2022). A Systematic Review of Ovarian Tissue Transplantation Outcomes by Ovarian Tissue Processing Size for Cryopreservation. Front. Endocrinol..

[10] Hossay C., Donnez J., Dolmans M.M. (2020). Whole Ovary Cryopreservation and Transplantation: A Systematic Review of Challenges and Research Developments in Animal Experiments and Humans. J. Clin. Med..

[11] De Roo C., Tilleman K. (2021). In Vitro Maturation of Oocytes Retrieved from Ovarian Tissue: Outcomes from Current Approaches and Future Perspectives. J. Clin. Med..

[12] Kometas M., Christman G.M., Kramer J., Rhoton-Vlasak A. (2021). Methods of Ovarian Tissue Cryopreservation: Is Vitrification Superior to Slow Freezing?-Ovarian Tissue Freezing Methods. Reprod. Sci..

[13] Khattak H., Malhas R., Craciunas L., Afifi Y., Amorim C.A., Fishel S., Silber S., Gook D., Demeestere I., Bystrova O. (2022). Fresh and cryopreserved ovarian tissue transplantation for preserving reproductive and endocrine function: A systematic review and individual patient data meta-analysis. Hum. Reprod. Update.

[14] Behl S., Joshi V.B., Larson N.B., Young M.C., Bilal M., Walker D.L., Khan Z., Granberg C.F., Chattha A., Zhao Y. (2023). Vitrification versus slow freezing of human ovarian tissue: A systematic review and meta-analysis of histological outcomes. J. Assist. Reprod. Genet..

[15] Silber S.J., DeRosa M., Goldsmith S., Fan Y., Castleman L., Melnick J. (2018). Cryopreservation and transplantation of ovarian tissue: Results from one center in the USA. J. Assist. Reprod. Genet..

[16] Sheshpari S., Shahnazi M., Mobarak H., Ahmadian S., Bedate A.M., Nariman-Saleh-Fam Z., Nouri M., Rahbarghazi R., Mahdipour M. (2019). Ovarian function and reproductive outcome after ovarian tissue transplantation: A systematic review. J. Transl. Med..

[17] Xie B., Li J., Huang Y., Hang F., Hu Q., Yu J., Qin A. (2023). Assessing the impact of transplant site on ovarian tissue transplantation: A single-arm meta-analysis. Reprod. Biol. Endocrinol..

[18] Bahroudi Z., Zarnaghi M.R., Izadpanah M., Abedelahi A., Niknafs B., Nasrabadi H.T., Seghinsara A.M. (2022). Review of ovarian tissue cryopreservation techniques for fertility preservation. J. Gynecol. Obstet. Hum. Reprod..

[19] Silber S., Kagawa N., Kuwayama M., Gosden R. (2010). Duration of fertility after fresh and frozen ovary transplantation. Fertil. Steril..

[20] Dolmans M.M., von Wolff M., Poirot C., Diaz-Garcia C., Cacciottola L., Boissel N., Liebenthron J., Pellicer A., Donnez J., Andersen C.Y. (2021). Transplantation of cryopreserved ovarian tissue in a series of 285 women: A review of five leading European centers. Fertil. Steril..

[21] Telfer E.E., Andersen C.Y. (2021). In vitro growth and maturation of primordial follicles and immature oocytes. Fertil. Steril..

[22] Affdal A.O., Salama M., Ravitsky V. (2024). Ethical, legal, social, and policy issues of ovarian tissue cryopreservation in prepubertal girls: A critical interpretive review. J. Assist. Reprod. Genet..

[23] Eijkenboom L., Saedt E., Zietse C., Braat D., Beerendonk C., Peek R. (2022). Strategies to safely use cryopreserved ovarian tissue to restore fertility after cancer: A systematic review. Reprod. Biomed. Online.

[24] Vitale F., Dolmans M.-M. (2024). Comprehensive Review of In Vitro Human Follicle Development for Fertility Restoration: Recent Achievements, Current Challenges, and Future Optimization Strategies. J. Clin. Med..

[25] Yang Q., Zhu L., Jin L. (2020). Human Follicle In Vitro Culture Including Activation, Growth, and Maturation: A Review of Research Progress. Front. Endocrinol..

[26] Xiao S., Zhang J., Romero M.M., Smith K.N., Shea L.D., Woodruff T.K. (2015). In Vitro follicle growth supports human oocyte meiotic maturation. Sci. Rep..

[27] Wang W., Todorov P., Isachenko E., Rahimi G., Mallmann P., Wang M., Isachenko V. (2021). In Vitro activation of cryopreserved ovarian tissue: A single-arm meta-analysis and systematic review. Eur. J. Obstet. Gynecol. Reprod. Biol..

[28] Mohd Faizal A., Sugishita Y., Suzuki-Takahashi Y., Iwahata H., Takae S., Horage-Okutsu Y., Suzuki N. (2022). Twenty-first century oocyte cryopreservation-in vitro maturation of immature oocytes from ovarian tissue cryopreservation in cancer patients: A systematic review. Womens Health.

[29] Vuković P., Kasum M., Orešković D., Čehić E., Raguž J., Elezaj S., Beketić-Orešković L. (2019). Importance of ovarian tissue cryopreservation in fertility preservation and anti-aging treatment. Gynecol. Endocrinol..

[30] Anderson R., Fauser B. (2018). Ovarian tissue transplantation for hormone replacement. Reprod. Biomed. Online.

[31] Gullo G., Etrusco A., Cucinella G., Basile G., Fabio M., Perino A., De Tommasi O., Buzzaccarini G., Morreale C., Marchi L. (2022). Ovarian tissue cryopreservation and transplantation in menopause: New perspective of therapy in postmenopausal women and the importance of ethical and legal frameworks. Eur. Rev. Med. Pharmacol. Sci..

[32] Fabbri R., Vicenti R., Magnani V., Paradisi R., Lima M., De Meis L., Rossi S., Raimondo D., Casadio P., Venturoli S. (2022). Ovarian tissue cryopreservation and transplantation: 20 years experience in Bologna University. Front. Endocrinol..

[33] Van der Ven H., Liebenthron J., Beckmann M., Toth B., Korell M., Krüssel J., Frambach T., Kupka M., Hohl M.K., Winkler-Crepaz K. (2016). Ninety-five orthotopic transplantations in 74 women of ovarian tissue after cytotoxic treatment in a fertility preservation network: Tissue activity, pregnancy and delivery rates. Hum. Reprod..

[34] Segers I., Bardhi E., Mateizel I., Van Moer E., Schots R., Verheyen G., Tournaye H., De Vos M. (2020). Live births following fertility preservation using in-vitro maturation of ovarian tissue oocytes. Hum. Reprod..

[35] Jeve Y.B., Gelbaya T., Fatum M. (2019). Time to consider ovarian tissue cryopreservation for girls with Turner’s syndrome: An opinion paper. Hum. Reprod. Open.

[36] Chaudhri E.N., Salman A., Awartani K., Khan Z., Hashmi S.K. (2024). Ovarian Tissue Cryopreservation versus Other Fertility Techniques for Chemoradiation-Induced Premature Ovarian Insufficiency in Women: A Systematic Review and Future Directions. Life.

[37] Fraison E., Huberlant S., Labrune E., Cavalieri M., Montagut M., Brugnon F., Courbiere B. (2023). Live birth rate after female fertility preservation for cancer or haematopoietic stem cell transplantation: A systematic review and meta-analysis of the three main techniques; embryo, oocyte and ovarian tissue cryopreservation. Hum. Reprod..

[38] Varlas V.N., Bors R.G., Albu D., Penes O.N., Nasui B.A., Mehedintu C., Pop A.L. (2021). Social Freezing: Pressing Pause on Fertility. Int. J. Environ. Res. Public Health.

[39] Gillipelli S.R., Pio L., Losty P.D., Abdelhafeez A.H. (2024). Female Fertility Cryopreservation Outcomes in Childhood Cancer: A Systematic Review. J. Pediatr. Surg..

